# Investigating the Polymorphism of Bone Morphogenetic Protein Receptor-1B (*BMPR1B*) Gene in Markhoz Goat Breed

**DOI:** 10.3390/ani10091582

**Published:** 2020-09-04

**Authors:** Sheila Pourali Dogaheh, Seyed Ziaeddin Mirhoseini, Vincenzo Tufarelli, Navid Ghavi Hossein-Zadeh, Sajad Badbarin, Maria Antonietta Colonna, Alireza Seidavi, Maria Selvaggi

**Affiliations:** 1 Department of Animal Science, Faculty of Agricultural Sciences, University of Guilan, Rasht 4199613776, Iran; sheilla.pourali@gmail.com (S.P.D.); mirhosin@guilan.ac.ir (S.Z.M.); nhosseinzadeh@guilan.ac.ir (N.G.H.-Z.); 2 Department of DETO, Section of Veterinary Science and Animal Production, University of Bari “Aldo Moro”, Valenzano, 70010 Bari, Italy; 3 Animal Science Research Department, Kermanshah Agricultural and Natural Resources Research and Education Center, AREEO, Kermanshah 67146, Iran; badbarin1688@yahoo.com; 4 Department of Agricultural and Environmental Science, University of Bari “Aldo Moro”, 70121 Bari, Italy; mariaantonietta.colonna@uniba.it (M.A.C.); maria.selvaggi@uniba.it (M.S.); 5 Department of Animal Science, Rasht Branch, Islamic Azad University, Rasht 4147654919, Iran; alirezaseidavi@iaurasht.ac.ir

**Keywords:** Markhoz goat, *BMPR1B*, PCR-RFLP, fecundity, twinning

## Abstract

**Simple Summary:**

The *BMPR1B* gene is one of the major genes involved in controlling prolificacy in small ruminant species. The research was conducted to detect possible polymorphisms in *BMPR1B* gene in a population of Markhoz goats, a valuable genetic resource of Iran. The results showed that all the analyzed individuals did not carry the previously reported FecB mutant allele. Moreover, we reported for the first time two novel possible mutations in exon 8 of *BMPR1B* gene that are noteworthy of further investigation.

**Abstract:**

Reproductive traits in livestock species are genetically controlled by the action of single genes with a major effect, commonly known as fecundity genes. One of the genes involved in controlling prolificacy is *BMPR1B* (*FecB*), a dominant autosomal gene located in chromosome 6 responsible for the fecundity and twinning rate in sheep and goat species. Markhoz goat is a valuable Iranian genetic resource endangered by extinction. Increasing the genetic variability and reproductive performances of Markhoz goat could preserve and enhance its economic value. This study was carried out to detect possible polymorphisms in *BMPR1B* gene in a sample of 100 Markhoz goats from Iran. DNA samples were screened by PCR–RFLP to assess the presence of the previously reported FecB mutation. Finally, the amplicons from seven goats out of the 100 samples were sequenced. The results showed that all the analyzed individuals did not carry the previously reported FecB mutant allele. However, our findings revealed two novel possible mutations in exon 8 of *BMPR1B* gene (775A > G and 777G > A) that need further investigations.

## 1. Introduction

Genetic studies indicate that prolificacy traits in livestock species are genetically controlled by the action of single genes with a major effect (commonly known as fecundity genes) [1,2,3]. These genes involved in controlling prolificacy have been initially identified in sheep: *BMP15* [2,3], *BMPR1B* [4], and *GDF9* [2]. All these genes belong to the TGF-β superfamily (transforming growth factor beta) [5], playing a key role in the process of embryo development, ovulation rate and litter size. *BMPR1B* (bone morphogenetic protein receptor-1B) is a dominant autosomal gene located in chromosome 6 responsible for the fecundity and twinning in sheep and goat [6,7,8]. It was firstly identified in Booroola Merino sheep [9]. In particular, the A→G transition localized in exon 8 corresponding to the nucleotide 830 of the mature mRNA is known as *FecB* or Booroola gene. This mutation seems to arise from Garole sheep, a northeast Indian breed, and it was introduced in Australia in 1792 [10]; later, the mutation was spread in many sheep breeds all over the world. *BMPR1B*, expressed in oocytes and granulosa cells, has additive effect in follicular development and granulosa cells in ovary and it affects the production of 1.5 oocytes per estrus cycle in sheep and, consequently, it increases the twinning and litter size in the population [6,11]. BMPR1B is known as a receptor for different bone growth factors (BMP). In particular, the BMP 2, 4, and 6 inhibit both the baseline of FSH and the FSH-induced progesterone production in granulosa cells of small antral follicle stage in sheep. BMP 2, 4, 6, 7, and 15 are expressed in the ovary and they are considered as candidate ligands of the BMPR1B receptor [12,13,14,15].

Markhoz goat breed represents an important Iranian genetic resource, well adapted to arid and semi-arid environmental conditions, reared as multipurpose animals for milk, kids, hair, and hide production [16]. Markhoz goat has a coat called mohair which represents the main economic income for goat producers [17]. Reports show that the twinning rate in this breed is about 25%, and even triplet kids have been described [18]. Considering that this goat is facing the threat of extinction, to improve productive and reproductive traits could prevent the annihilation of its genetic heritage for the future [19]. Therefore, the objective of this study was to investigate the polymorphisms of *BMPR1B* gene in Markhoz goat breed and a possible association with prolificacy rate.

## 2. Materials and Methods

### 2.1. DNA Samples

In accordance to the International Guidelines for Research involving animals (Directive No. 2010/63/EU), there was no need to request the ethical approval; blood samples were made during the normal and routine checks for animal health status carried out by the official veterinarian of the farm.

Blood samples were collected from 100 Markhoz goats (males and females) progeny of eight different unrelated bucks and 65 dams. The number of paternal half-sibs/sisters varied from 4 to 8. The animals selected to sample were individuals with parents less genetically related. Samples were transported to the Laboratory of Molecular Genetics at the Faculty of Agricultural Sciences, University of Guilan, Iran, and stored at −20 °C until use. Individual whole blood samples were processed to extract genomic DNA by using modified saluting out method and suspended in TE buffer (1 mmol/L EDTA pH 8.0, 10 mmol/L Tris–HCl pH 8.0) and stored at −20 °C. DNA concentration and purity was spectrophotometrically determined calculating the ratio of optical densities at 260 nm and 280 nm.

### 2.2. Primers and PCR Amplification

The primers for detecting FecB mutation of *BMPR1B* gene were the same used by Dutta et al. [20]. According to the goat *BMPR1B* DNA sequence (GenBank accession number: AF357007, Gene ID: 443454), a 140 bp fragment, spanning exon 8 and flanking region, was amplified by using the following primers:

F: 5′-GTCGCTATGGGGAAGTTTGGATG-3′

R: 5′-CAAGATGTTTTCATGCCTCATCAACACGGTC-3′.

The reverse primer, deliberately introduced by a point mutation, would create an AvaII restriction site (G↓GACC) in amplicons from *FecB* carrier goat. The PCR was performed in 25 μL volume containing ~2.5 μmol/L of each primer, 2.5 μL of PCR buffer (10 mmol/L Tris–HCl pH 8.0, 50 mmol/L KCl, 0.1% Triton X-100), 3.75 mmol/L MgCl_2_, 0.1 mmol/L dNTP, 100 ng/μL caprine genomic DNA, 0.5 U Taq DNA polymerase (Promega, Madison, WI, USA), and the rest was distilled water. The touchdown PCR protocol was as follows: initial denaturation (95 °C for 5 min); followed by 33 cycles of denaturation (95 °C for 30 s), annealing (60 °C for 40 s), extension (72 °C for 1 min) on Mastercycler^®^ 5333 (Eppendorf AG, Hamburg, Germany) and a final extension cycle (72 °C for 5 min).

### 2.3. PCR-RFLP Analysis

PCR products of *BMPR1B* gene were digested at 37 °C for 3 h using AvaII restriction enzyme (New England Biolab, Ipswich, MA, USA). The digested amplicons were analyzed in 2.5% (*w*/*v*) agarose gel. The digested fragments were UV light visualized using a trans-illuminator to assess the banding pattern of the endonuclease cut products. The enzyme potentially cuts the amplicon into two fragments (110 and 30 bp) for the *FecB* carrier goat, while non carrier goat’s amplicon remains undigested (+). The heterozygotes will show three bands (140, 110 and 30 bp). So, we have to expect three different genotypes (*FecB*/*FecB*, *FecB*/+ and ++).

### 2.4. DNA Sequencing

To validate the PCR-RFLP results, the amplicons from seven goats out of the 100 samples were sequenced by automated DNA sequencer (ABI Genetic Analyzer, Foster City, CA, USA). The seven samples used for sequencing were from animals’ progeny of seven different bucks and does much less genetically related each other. The sequences were analyzed and compared using the Chroma software and sequence alignments were performed by using Mega 5 software [21].

## 3. Results

**Figure 1:**
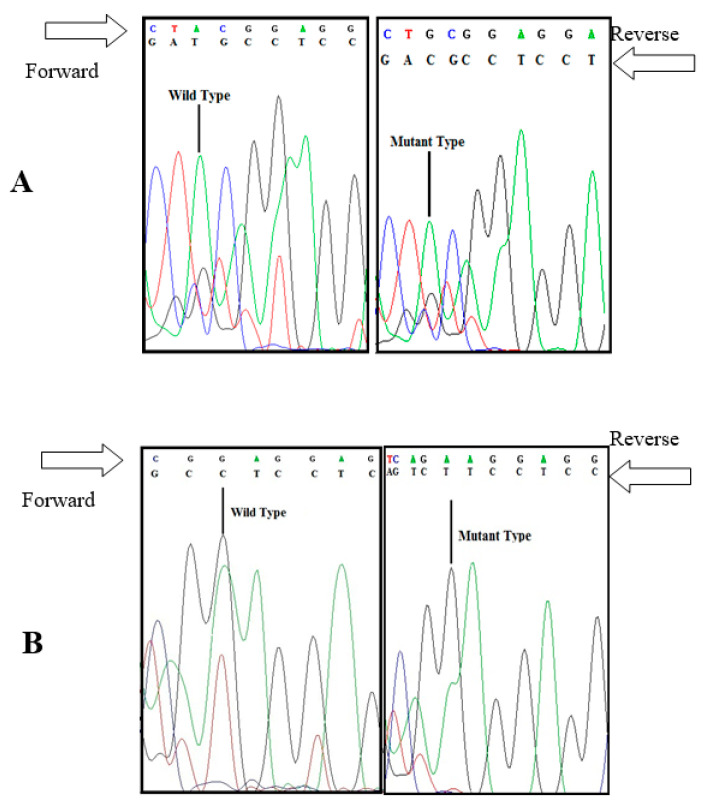
Detected mutations in exon 8 of *BMPR1B* gene of Markhoz goat breed. (**A**) Single nucleotide polymorphism 775A > G; (**B**) Single nucleotide polymorphism 777G > A.

**Figure 2:**
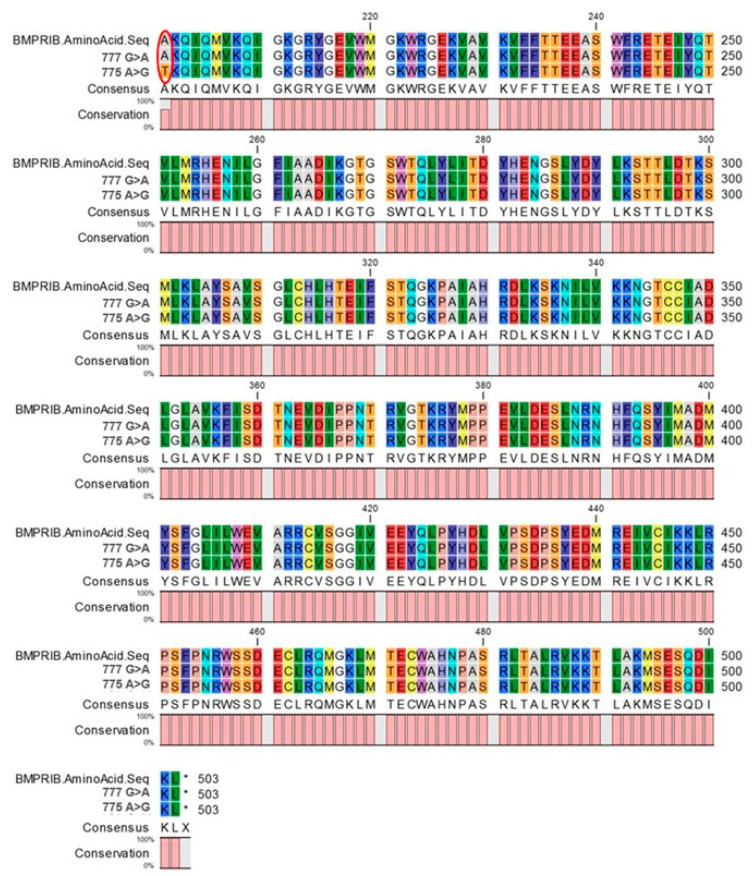
Polypeptide changes in presence of SNPs in *BMPR1B* gene in Markhoz goat breed (CLC Main workbench.7.7.1 version).

As expected, the 140 bp fragment of FecB was amplified without any specific band of unwanted products. The results of visible bands showed that AvaII enzyme did not recognize the restriction site. Therefore, the genotypes of all the 100 samples were ++. Complete concordance between PCR-RFLP results was obtained by sequence analysis of the amplicons, confirming no polymorphism at FecB locus in Markhoz goats. However, our findings revealed two novel possible mutations in exon 8 of *BMPR1B* gene: 775A > G and 777G > A. Both these mutations were found in three animals out of seven. The first one (775A > G) caused an amino acid change in the mature protein chain (Figure 1), which is responsible of a replacement of a Threonine instead of Alanine at position 201 (A201T) (Figure 2). The 777G > A mutation did not cause amino acid change.

## 4. Discussion

Different studies revealed that the mutations in fecundity genes are associated with both ovulation rate and litter size of sheep [4,6,7,9,11,22]. The identification of the FecB mutation in *BMPR1B* gene, initially discovered in Merino Booroola sheep, is important for small ruminant production. Subsequently, the presence of FecB mutation was identified also in other sheep breeds worldwide, such as: Garole, Javanese, Han, and Hu [10,23,24,25,26,27,28,29]. On the other side, previous investigations in different sheep breeds revealed a lack of FecB mutation [30,31]. The FecB mutation has been investigated also in many goat breeds. Recently, Ahlawat et al. [32] studied the FecB mutation in goats belonging to six prolific breeds (Black Bengal, Beetal, Barbari, Malabari, Sikkim, and Jakhrana) and found them to be homozygous non-carriers. The absence of FecB mutation was also reported by Chu et al. [33] in five native goat breeds from China, and by Hua et al. [34] in four Chinese goat breeds. Moreover, Palai et al. [35] studied FecB polymorphism in Raighar goat and reported the monomorphic status of this breed. Conversely, Shokrollahi and Morammazi [36] discovered that the *BMPR1B* gene was polymorphic in Markhoz goat with a frequency of FecB homozygotes of 0.4939, although no significant effect of litter size in does was found. A possible explanation for this discrepancy between results obtained from two different flocks of the same breed could be the structure of the analyzed population and a possible previous gene introgression as breeding strategy. Furthermore, Polley et al. [1] reported the presence of all three possible genotypes at FecB locus in Black Bengal goat breed, being the FecB/+ genotype the most frequent.

Considering that Markhoz goat breed of Iran is reared under harsh environmental conditions, this aspect may have determined a natural selection pressure against prolificacy traits, leading to a low rate of kids per doe. In fact, twinning rate is ruled by both environmental and genetic factors (mainly feed availability and plane of nutrition), and it is currently known that the difficult environmental conditions and twinning are in conflict each other [37]. Moreover, the introgression of the FecB allele from cosmopolitan breeds to the Markhoz is improbable considering the geographical and reproductive isolation of this goat population. Thus, to undertake more studies on a larger sample, also to clarify the role of the two novel possible mutations detected in the present population, is compulsory.

## 5. Conclusions

In the present study, we reported the absence of *FecB* mutation in *BMPR1B* gene in Markhoz goat breed. Interestingly, two novel possible mutations have been firstly reported that may be worthy of further investigations in order to clarify the role of *BMPR1B* gene on ovulation rate and litter size in this breed. Thus, it is possible to suppose that Markhoz goat litter size is not ascribable to *FecB* allele and may be probably influenced by other major genes; in fact, other investigations showed that *FecB* allele might be not the only cause for prolificacy rate in goat.
